# Ethanol Extract of* Mylabris phalerata* Inhibits M2 Polarization Induced by Recombinant IL-4 and IL-13 in Murine Macrophages

**DOI:** 10.1155/2017/4218468

**Published:** 2017-07-25

**Authors:** Hwan-Suck Chung, Bong-Seon Lee, Jin Yeul Ma

**Affiliations:** Korean Medicine (KM) Application Center, Korea Institute of Oriental Medicine (KIOM), 70 Cheomdan-ro, Dong-gu, Daegu 41062, Republic of Korea

## Abstract

*Mylabris phalerata* (MP) is an insect used in oriental herbal treatments for tumor, tinea infections, and stroke. Recent studies have shown that tumor-associated macrophages (TAM) have detrimental roles such as tumor progression, angiogenesis, and metastasis. Although TAM has phenotypes and characteristics in common with M2-polarized macrophages, M1 macrophages have tumor suppression and immune stimulation effects. Medicines polarizing macrophages to M1 have been suggested to have anticancer effects via the modulation of the tumor microenvironment. In this line, we screened oriental medicines to find M1 polarizing medicines in M2-polarized macrophages. Among approximately 400 types of oriental medicine, the ethanol extract of* M. phalerata* (EMP) was the most proficient in increasing TNF-*α* secretion in M2-polarized macrophages and TAM. Although EMP enhanced the levels of an M1 cytokine (TNF-*α*) and a marker (CD86), it significantly reduced the levels of an M2 marker (arginase-1) in M2-polarized macrophages. In addition, EMP-treated macrophages increased the levels of M1 markers (*Inos* and* Tnf-α*) and reduced those of the enhanced M2 markers (*Fizz-1, Ym-1,* and* arginase-1*). EMP-treated macrophages significantly reduced Lewis lung carcinoma cell migration in a transwell migration assay and inhibited EL4-luc2 lymphoma proliferation. In our mechanism study, EMP was found to inhibit STAT3 phosphorylation in M2-polarized macrophages. These results suggest that EMP is effective in treating TAM-mediated tumor progression and metastasis.

## 1. Introduction

Tumor burden comprises a group of heterogeneous cells, including T cells, neutrophils, and macrophages. The major cells comprising the tumor burden are macrophages, accounting for approximately 50% of the burden. Tumor-associated macrophages (TAM) are involved in tumor progression and metastasis, and the number of TAM in the tumor burden is positively correlated with poor prognosis [1]. Macrophages can be M1 polarized by stimulation with IFN-*γ* or LPS, and these M1-polarized macrophages secrete IL-12, TNF-*α*, and IL-1*β*, which kill cancer cells [2]. However, M2 macrophages are polarized by stimulation with IL-4, IL-13, or M-CSF and release IL-10, CCL17, and CCL22, which help in tumor progression and metastasis. M2 macrophages have phenotypes and functions similar to TAM [3, 4]. Because TAM play a critical role in tumor progression and metastasis, many researchers have studied the control of TAM and have shown that switching TAM or M2 with M1 significantly inhibits tumor progression and metastasis [5–7]. Therefore, switching TAM or M2 with M1 is a potential target for cancer treatment.

*Mylabris phalerata* (MP) is an insect used in the preparation of Mylabris, a Korean medicine listed in the Korean Herbal Pharmacopoeia that is used to treat tumors. MP has antitumor effects and a proliferative effect on leucocytes [8, 9]. The major component of MP, cantharidin, also has anticancer and apoptotic effects on cancer cells [10]. Norcantharidin, a demethylated form of cantharidin, is used as an anticancer drug in China [11].

We screened 400 herbal ethanol extracts to examine the effect of M1 polarization on M2-polarized macrophages induced by IL-4 and IL-13. We found that the ethanol extract of MP (EMP) polarized M2 into M1 and that this effect was not mediated by endotoxins.

## 2. Materials and Methods

### 2.1. Bone Marrow Macrophage Culture

Animal procedures were approved by the IACUC in the Korea Institute of Oriental Medicine. Bone marrow cells (BMC) were isolated from the tibia and femur of 6-week-old male C57BL/6 mice (Samtako Bio Korea, Gyeonggi-do, South Korea). Bone marrow macrophages (BMM) generated using BMC were differentiated in the RPMI1640 medium supplemented with 10% FBS and macrophage colony-stimulating factors (M-CSF, 60 ng/mL, Peprotech, Rocky Hill, NJ, USA) for 1 week. The medium was replaced with a fresh M-CSF-containing medium 3 days after seeding the cells.

### 2.2. TAM Culture

To prepare TAM, mice were subcutaneously implanted with Lewis lung carcinoma (LLC) cells (2 × 10^5^/mouse). They were sacrificed after 3 weeks, and tumor tissues were isolated. Single cells were dissociated from tumor tissues using a tumor dissociation kit (cat. 130-096-730, Miltenyi Biotec, Bergisch Gladbach, Germany) following the manufacturer's instructions. To separate the macrophages, the cells were labelled with CD11b microbeads (cat. 130-049-601, Miltenyi Biotec), and the CD11b+ cells (macrophages) were isolated with MACS columns. Approximately 10%–20% of the tumor-dissociated cells were CD11b+. When we analyzed the purity of TAM, over 90% were CD11b+.

### 2.3. Preparation of EMP

MP was purchased from an herbal supplier (Yeongcheon herb, Yeongcheon, Korea), and a voucher specimen (number E233) was deposited in the herbal bank of the Korea Medicine Application Center, Korea Institute of Oriental Medicine. To prepare EMP, dried MP (30 g) was ground into a fine powder, soaked in 300 mL of 70% ethanol, and extracted in a shaking incubator at 40°C for 24 h. The extract was filtered through a testing sieve (150 *μ*m; Retsch, Haan, Germany), evaporated on a rotary evaporator, concentrated by lyophilization, and then stored at −20°C. EMP powder (50 mg) was dissolved in 10 mL of 50% ethanol (v/v) and filtered through a 0.22 *μ*m disk filter. Endotoxin was examined using the Pierce LAL chromogenic endotoxin quantitation kit (Thermo Scientific, Bonn, Germany) according to the manufacturer's protocol.

### 2.4. TNF-*α* and TGF-*β* Analysis

To polarize BMM to M2, they were treated with recombinant IL-4 (20 ng/mL) and IL-13 (20 ng/mL) for 6 h. EMP was added for 18 h, and the supernatants were harvested and kept at −80°C until use. TNF-*α* and TGF-*β* were analyzed by OptEIA ELISA kit (BD Biosciences Pharmingen, San Diego, CA, USA) and eBioscience™ Human/Mouse TGF beta 1 ELISA Ready-SET-Go!™ Kit, 2nd Generation (cat. 88-8350-76, eBioscience, San Diego, CA, USA), respectively, following manufacturer's instruction.

### 2.5. Real-Time PCR

To polarize BMM to M2, they were treated with recombinant IL-4 (20 ng/mL) and IL-13 (20 ng/mL) for 6 h. EMP was added for 18 h, and the cells were harvested. Total RNA was extracted using the EasyBlue RNA extraction kit (iNtRON Biotechnology, Inc., Seongnam, Korea). The quality and concentration of the RNA were assayed using the ND-1000 spectrophotometer (Nanodrop Technologies, Wilmington, DE, USA). cDNA was synthesized using CycleScript Reverse Transcriptase (Bioneer, Seoul, Korea) and stored at −20°C. Real-time PCR was conducted using the CFX96 Touch Real-Time PCR System (Bio-Rad, CA, USA) employing the AccuPower GreenStar qPCR Master Mix (Bioneer, Daejeon, Korea). The PCR protocol comprised 10 min at 95°C followed by 45 cycles of 10 s at 95°C, 10 s at 60°C, and 10 s at 72°C. After the cycles were completed, the signal at each temperature between 65°C and 95°C was recorded to generate a dissociation curve. The sequences of the murine primers were listed in Table 1. The target mRNA levels were compared by calculating the crossing point (Cp) value and normalized to the reference gene* GAPDH*.

### 2.6. Preparation of Nuclear and Cytoplasmic Extracts

RAW264.7 cells (5 × 10^6^) were pretreated with IL-4 (20 ng/mL) + IL-13 (20 ng/mL) for 6 h to polarize to M2 and then treated with EMP for 1 h. Nuclear and cytoplasmic extracts were prepared using NE-PER Nuclear and Cytoplasmic Extraction Reagents (Thermo Fisher Scientific, Rockford, IL, USA) according to the manufacturer's protocol.

### 2.7. Western Blotting

Cells were washed with phosphate-buffered saline (PBS) and lysed using the radioimmunoprecipitation assay buffer (Millipore, MA, USA) containing protease and phosphatase inhibitors. Total protein (15–20 *μ*g) was separated by 10% SDS-PAGE gel electrophoresis, transferred to polyvinylidene fluoride (PVDF) membrane, and immunoblotted with specific antibody. Antibodies for arginase-1, *β*-actin (Santa Cruz Biotechnology, CA, USA), phosphorylated signal transducer and activator of transcription 3 (p-STAT3) (Tyr705), p-STAT6 (Tyr641), P65, p-i*κ*B-*α*, and proliferating cell nuclear antigen (PCNA) (Cell Singling Technology, MA, USA) were used in this study. Chemiluminescent signals were detected using the ChemiDoc imaging system (Bio-Rad Laboratories, CA, USA) and a chemiluminescence reagent (Thermo Scientific, Rockford, IL, USA).

### 2.8. LLC Tumor Cell Migration Assay

Cell migration was assayed using a 24-transwell chamber with a diameter of 6.5 mm and an 8 *μ*m pore polyethylene terephthalate (PET) membrane (SPL Lifesciences, Seoul, Korea) as described by Kim et al. [12].

### 2.9. Tumor Cell Proliferation Assay

EL4-luc2 cells, a lymphoma cell line from C57BL/6 mice expressing the firefly luciferase gene (Caliper Life Science, MA, USA), were used to evaluate drug efficacy on macrophage tumoricidal activity in coculture conditions. BMM were pretreated with IL-4 (20 ng/mL) + IL-13 (20 ng/mL) for 6 h to polarize to M2 and then with EMP for 18 h. The cells were washed with DPBS and 3 × 10^4^ cells in 200 *μ*l of media were seeded in 96-well white plates. EL4-luc2 cells (1 × 10^4^ cells) were cocultured with BMM for 48 h. Luciferin (150 *μ*g/mL) was added and luminescence was detected using the SpectraMax L microplate reader (Molecular Devices, Sunnyvale, CA, USA).

### 2.10. Statistical Analysis

All values are expressed as means ± SEM. The statistical significance (*p* < 0.05 for all analyses) was assessed by one-way ANOVA followed by Tukey's post hoc test for multiple comparisons using the Prism 5.01 software (GraphPad Software Inc., San Diego, CA, USA).

## 3. Results

### 3.1. Increased TNF-*α* and Decreased TGF-*β* Release by EMP in M2 Macrophages and TAM

**Figure 1:**
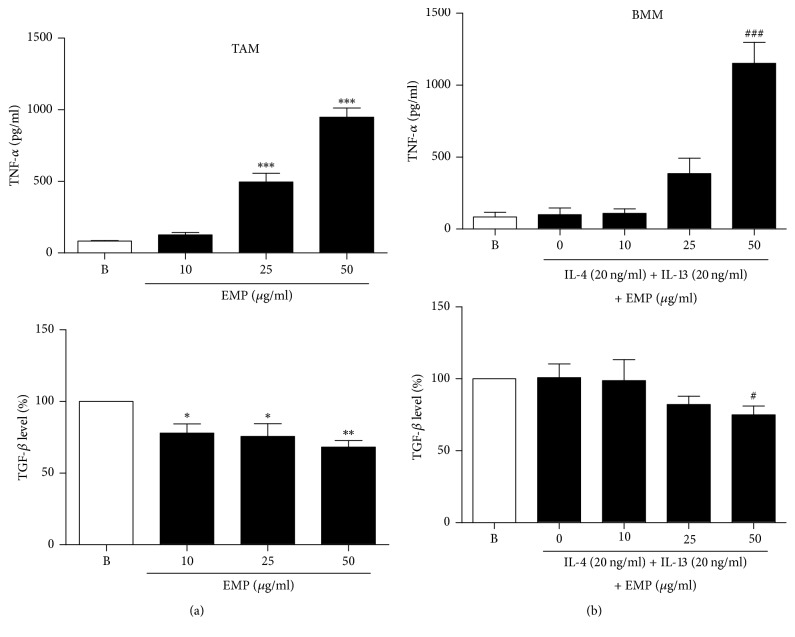
*Increased TNF-α and decreased TGF-β in M2 macrophages and TAM by treatment with EMP*. (a) TAM were isolated with CD11b microbeads after dissociation of tumor tissue into single cells. CD11b^+^ TAM were treated with various doses of EMP for 24 h. (b) BMM were pretreated with IL-4 (20 ng/mL) + IL-13 (20 ng/mL) for 6 h to polarize to M2 and then EMP was added for 18 h. TNF-*α* and TGF-*β* release were assessed by ELISA. Values are indicated as mean ± SEM. ^*∗*^*p* < 0.05, ^*∗∗*^*p* < 0.01, and ^*∗∗∗*^*p* < 0.001, compared with the B (Blank) samples; ^#^*p* < 0.05 and ^###^*p* < 0.001, compared with the 0 (EMP nontreated) samples.

Because TNF-*α* is a prominent M1 marker, we screened 400 types of herbal extracts for their effect on TNF-*α* release in M2 macrophages. M2 macrophages were induced by treating BMM with mouse recombinant IL-4 (20 ng/mL) and IL-13 (20 ng/mL) for 6 h, after which EMP was added for 18 h. Among the 400 herbal extracts, EMP showed the strongest effect on TNF-*α* release in M2 macrophages. TNF-*α* induction in M2 macrophages by EMP was also shown in TAM (Figure 1). On the other hand, TGF-*β* is a typical M2 marker. TGF-*β* release was reduced by EMP treatment in TAM and BMM (Figure 1). To exclude the possibility of TNF-*α* release by endotoxin contamination in EMP, we also examined the endotoxin level in EMP and found that it was less than 0.1 EU/mL (data not shown).

### 3.2. Enhanced M1 and Reduced M2 Markers after EMP Treatment in M2 Macrophages

**Figure 2:**
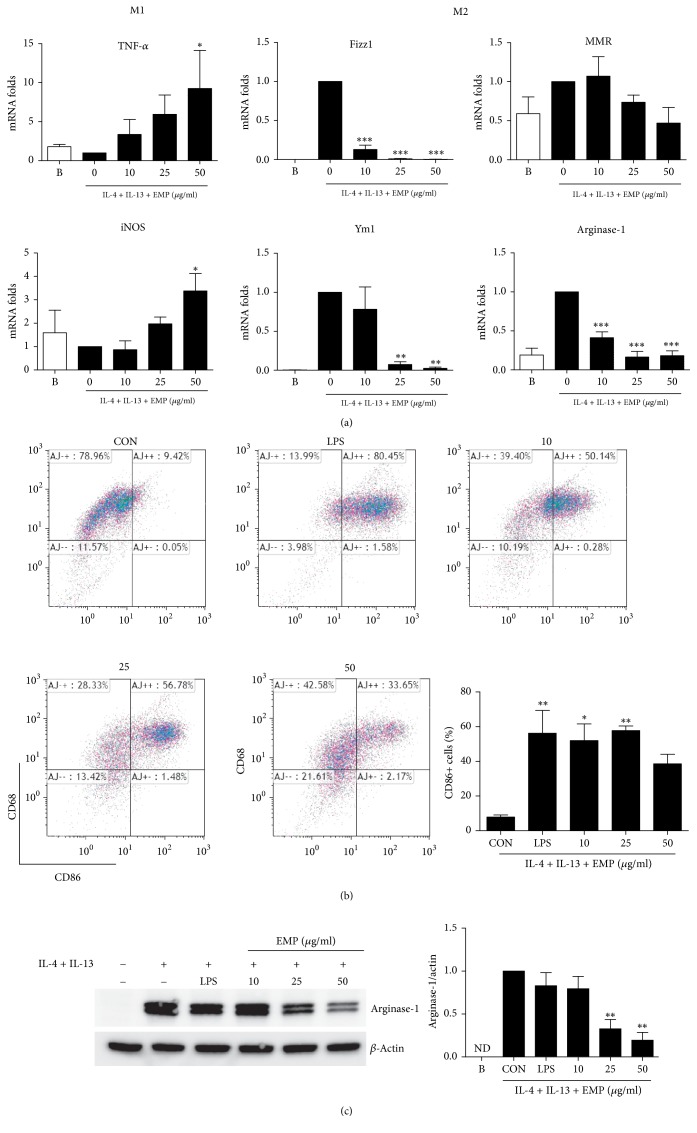
*Repolarization to M1 by EMP in M2 macrophages*. (a) Effect of EMP on mRNA expression in M2 macrophages. BMM were pretreated with IL-4 (20 ng/mL) + IL-13 (20 ng/mL) for 6 h to polarize to M2 and then EMP was added for 18 h. The amounts of mRNA were quantified by real-time RT-PCR. The expression levels of mRNA were normalized by dividing the values by the GAPDH intensity. (b) CD86+ and CD68+ BMM after EMP treatment were analyzed by flow cytometry. (C) Arginase-1 expression was determined by Western blotting. ND = not detected. Values are indicated as the mean ± SEM. ^*∗*^*p* < 0.05, ^*∗∗*^*p* < 0.01, and ^*∗∗∗*^*p* < 0.001 compared with the 0 (CON, EMP nontreated) samples.

We analyzed the expression of M1 and M2 genes after EMP treatment in M2 macrophages. Although the increased M2 markers (*Fizz1*,* Ym1*, and* Arg1*) were significantly inhibited by EMP, M1 (*Tnfa* and* Inos*) markers were significantly increased by EMP based on the real-time RT-PCR analysis (Figure 2(a)). When we analyzed M1 (CD86) and M2 (CD68) phenotype changes using flow cytometry after EMP treatment in M2 macrophages, EMP significantly increased CD86 expression but did not affect CD68 expression (Figure 2(b)). Arginase-1 catalyzes L-arginine as a substrate and produces L-ornithine and urea. It is known that the depletion of L-arginine by arginase-1 could inhibit the L-arginine-dependent immune functions [13]. For instance, L-arginine depletion suppresses T-cell proliferation [14]. Although macrophages polarized to M2 by IL-4 and IL-13 displayed a significantly increased expression of arginase-1, EMP alleviated the increased expression of arginase-1 in a dose-dependent manner. Intriguingly, LPS did not significantly alter the increased expression of arginase-1 (Figure 2(c)).

### 3.3. EMP-Treated Macrophages Attenuate LLC Tumor Migration and EL4-luc2 Lymphoma Proliferation

**Figure 3:**
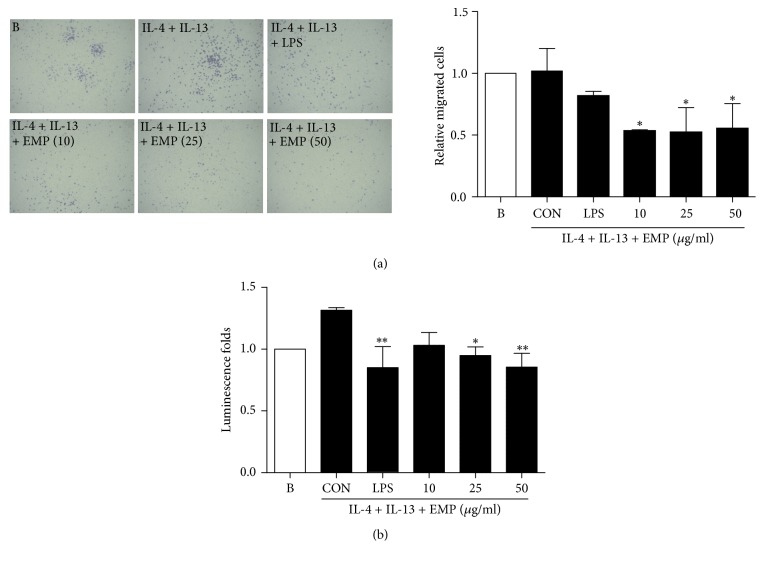
*EMP-treated macrophages reduce migration and proliferation of tumor cells*. (a) A transwell migration assay was performed to determine the migration of LLC tumor cells by EMP-treated macrophages. LLC tumor cells on the lower surface of the transwell membrane were stained with crystal violet solution and observed under a phase contrast microscope with 50x magnification. (b) EL4-luc2 lymphoma was cocultured with EMP-treated macrophages for 48 h. Values are indicated as the mean ± SEM. ^*∗*^*p* < 0.05 and ^*∗∗*^*p* < 0.01 compared with the CON samples.

We studied the effect of macrophages polarized by EMP on LLC tumor cell migration. To exclude a direct effect of EMP on LLC, EMP-treated macrophages were washed out with DPBS and the macrophages were seeded in the lower compartment and then LLC were cultured in the upper compartment of the transwell chamber. As shown in Figure 3(a), EMP treatment in M2 macrophages attenuated the migration of LLC tumor cells in a dose-dependent manner. We also evaluated the tumoricidal activity of EMP-treated macrophages in coculture conditions with EL4-luc2 lymphoma. LPS- and EMP-treated macrophages significantly reduced EL4-luc2 proliferation compared with the control group (Figure 3(b)). These data show that EMP-treated M2 macrophages can inhibit tumor metastasis and progression.

### 3.4. EMP Inhibits STAT3 Phosphorylation in M2 Macrophages

**Figure 4:**
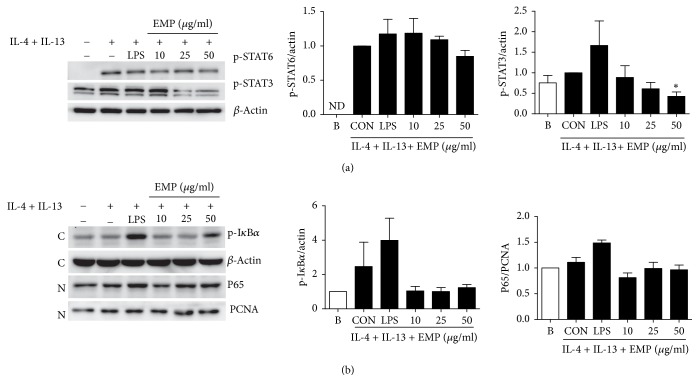
*Inhibition of STAT3 phosphorylation by EMP in M2 macrophages*. (a) BMM were pretreated with IL-4 (20 ng/mL) + IL-13 (20 ng/mL) for 6 h to polarize to M2 and then EMP was added for 1 h. The phosphorylation of STAT3 and STAT6 was analyzed by immunoblot analysis. ND = not detected. (b) RAW264.7 cells were pretreated with IL-4 (20 ng/mL) + IL-13 (20 ng/mL) for 6 h to polarize to M2 and then EMP was added for 1 h. p-I*κ*B*α* and *β*-actin were analyzed in the cytosolic fraction and P65 and PCNA were analyzed in the nuclear fraction. C = cytosol; N = nucleus. Values are indicated as the mean ± SEM. ^*∗*^*p* < 0.05 compared with the CON samples.

To study the mechanism of EMP in M2 macrophages, we analyzed STAT6 phosphorylation, which is a critical transcription factor in the M2 polarization induced by IL-4 and IL-13. Although M2 macrophages increased STAT6 phosphorylation, there were no significant differences in STAT6 phosphorylation upon EMP treatment (Figure 4(a)). NF-*κ*B is a critical transcription factor for proinflammatory cytokines such as TNF-*α*, IL-6, and IL-1*β*. Although LPS treatment increased the translocation of p65 (NF-*κ*B subunit) into the nucleus and the phosphorylation of I-*κ*B *α* in cytoplasm, EMP did not show any significant changes in NF-*κ*B translocation or I-*κ*B *α* phosphorylation (Figure 4(b)). It has been reported that STAT3 suppression can convert TAM's phenotype from M2 to M1 [15, 16]. We also explored whether STAT3 was involved in the effect of EMP on macrophage polarization. The phosphorylation of STAT3 induced by IL-4 and IL-13 was diminished by EMP treatment (Figure 4(a)).

## 4. Discussion

EMP enhances M1 cytokine (TNF-*α*) release and inhibits M2 cytokine (TGF-*β*) and M2 markersw such as YM1, Fizz1, and arginase-1 in M2 polarized macrophages by IL-4 and IL-13. In addition, EMP increases M1 phenotype CD86 expression and reduces M2 phenotype arginase-1 expression in M2-polarized macrophages. These data indicate that EMP can repolarize the M2-polarized macrophages into M1 macrophages. M1-polarized macrophages induced by EMP inhibited LLC cancer-cell migration and EL-4 lymphoma proliferation. Because STAT6 is a critical signal pathway of IL-4 and IL-13 [17], we studied the phosphorylation of STAT6 in M2 macrophages. Although the phosphorylation of STAT6 is remarkably increased in M2 macrophages, LPS and EMP did not affect the phosphorylation of STAT6 in M2 macrophages. In addition, we also explored the translocation of NF-*κ*B, a key transcription factor for inflammation, into the nucleus by EMP. Although the phosphorylation of I-*κ*B in cytoplasm and the translocation of p65 into the nucleus were increased by LPS, EMP did not affect the phosphorylation and translocation. Inhibition of STAT3 phosphorylation in a shift from M2 to M1 polarization has been reported to suppress the growth and metastasis of tumor cells [15, 16]. In the present study, we show that EMP inhibits STAT3 phosphorylation in M2 macrophages.

When we screened the TNF-*α* secretion in M2 macrophages using 400 species of herb,* Mylabris phalerata*, Genkwa Flos, Solani Nigri Herba, Pinelliae Tuber, Sambuci Lignum, Sanguisorbae Radix, Euphorbiae Kansui Radix, Phaseoli Radiati Semen, Poria, and Melandrii Herba were the most potent top 10 herbs without endotoxin. Although there are some reports on anti-inflammatory effects of these 10 herbs, macrophage polarization by these herbs has not been studied.

It has been reported that norcantharidin, a biosynthesized demethylated cantharidin, has anticancer effects by the regulation of M1 macrophage polarization via miR-214 expression [16]. Because norcantharidin is a synthetic compound and is not a component of MP, the effects of EMP on M1 polarization may not be mediated by norcantharidin.

There is a lot of evidence showing that M2-polarized macrophages can be converted to M1 macrophages and the converted M1 macrophages exert anticancer and antimetastatic properties [15, 18–20]. Although we did not perform an in vivo study, there are many reports on the anticancer effects of MP [8, 21, 22]. Because MP per se has anticancer effects, it may not be easy to differentiate its anticancer effects by tumor killing from those by M1 polarization. Conversely, it is supposed that the anticancer effects of MP in animal are mediated by M1 polarization and not just by the apoptosis of tumor cells.

These findings suggest that treatment with EMP polarizes M2/TAM into M1. Because of these effects of EMP, it may be used as an adjuvant for anticancer drugs to boost anticancer immunotherapy.

## Figures and Tables

**Table 1:** Primers used for real-time RT-PCR.

Target gene	Primer sequence
TNF-*α*	F: 5′-TTCTGTCTACTGAACTTCGGGGTGATCGGTCC-3′
	R: 5′-GTATGAGATAGCAAATCGGCTGACGGTGTGGG-3′

Fizz1	F: 5′-TGGAGAATAAGGTCAAGGAAC-3′
	R: 5′-GTCAACGAGTAAGCACAGG-3′

YM1	F: 5′-CATTCAGTCAGTTAT CAGATTCC-3′
	R: 5′-AGTGAGTAGCAGCCTTGG-3′

iNOS	F: 5′-GGCAGCCTGTGAGACCTTTG-3′
	R: 5′-GCATTGGAAGTGAAGCGTTTC-3′

Arginase 1	F: 5′-AGACAGCAGAGGAGGTGAAGAG-3′
	R: 5′-CGAAGCAAGCCAAGGTTAAAGC-3′

MMR	F: 5′-AGTGGCAGGTGGCTTATG-3′
	R: 5′-GGTTCAGGAGTTGTTGTG-3′

GAPDH	F: 5′-ACCCAGAAGACTGTGGATGG-3′
	R: 5′-CACATTGGGGGTAGGAACAC-3′

F, forward; R, reverse.
